# Evaluation of Visual and Optical Coherence Tomography Outcomes in Patients with Leber’s Hereditary Optic Neuropathy Treated with Idebenone

**DOI:** 10.3390/life15081172

**Published:** 2025-07-23

**Authors:** Raluca Eugenia Iorga, Andreea Dana Moraru, Răzvana Sorina Munteanu-Dănulescu, Delia Urdea, Ciprian Danielescu

**Affiliations:** 1 Department of Surgery II, Discipline of Ophthalmology, “Grigore T. Popa” University of Medicine and Pharmacy, Strada Universitatii No. 16, 700115 Iași, Romania; ralucadanulescu@yahoo.com (R.E.I.); ciprian.danielescu@umfiasi.ro (C.D.); 2 Department of Gastroenterology, “L. Pasteur” Clinical Hospital, 28630 Le Coudray, France; razvanadanulescu@yahoo.com; 3 Discipline of Alergology, Doctoral School “Grigore T. Popa” University of Medicine and Pharmacy, Strada Universitatii No. 16, 700115 Iași, Romania; deliaurdea@yahoo.com

**Keywords:** Leber hereditary optic neuropathy, idebenone, clinically relevant benefit, optic coherence tomography

## Abstract

The aim of this paper is to present our experience with the diagnosis and management of nine patients diagnosed with Leber’s hereditay optic neuropathy. Materials and methods: We conducted a prospective, observational study that included nine patients treated with idebenone, followed for a period of 18 months. Results: Our findings suggest that the impact of treatment varies significantly depending on the disease phase. In the acute phase, visual acuity deteriorated from 0.67 logMAR at onset to 0.97 logMAR at 3 months, followed by a slight improvement to 0.88 logMAR at 9 months. In the chronic phase, average values decreased progressively from 1.44 logMAR at onset to 1.26 logMAR at 12 and 18 months. We also observed a consistent treatment benefit over time in eyes harbouring the m.11778 G > A mutation. Although the most powerful predictor of visual outcome remains the mtDNA genotype, young age at onset is correlated with a better prognosis. In the acute phase, more cases of a clinically relevant benefit were observed than expected (33.33% versus 22.22% expected), and fewer clinically relevant worsening cases were observed (0% versus 11.11% expected). Regarding OCT measurement, our study highlighted a significant difference in peripapillary retinal nerve fiber layer thickness between the initial evaluation and the 6-month follow-up (100.83 µm ± 30.2 at baseline versus 96.7 µm ± 24.8 at 6 months). Conclusions: Our paper demonstrates the benefit of idebenone treatment in improving visual acuity in patients with Leber hereditary optic neuropathy. We highlighted the importance of long-term treatment, emphasizing that extended administration is key to achieving favorable outcomes.

## 1. Introduction

Hereditary optic neuropathies are a heterogeneous group of genetically determined diseases that affect the optic nerve [1]. The two most common inherited optic neuropathies are Leber’s hereditary optic neuropathy (LHON) and dominant optic atrophy (DOA).

Leber’s hereditary optic neuropathy (LHON) is a rare disease, with an estimated prevalence of 1 in 45,000 in the European population [2]. Rare diseases are defined as conditions affecting fewer than 1 in 2000 people in the general population [3]. LHON is one of the most commonly studied mitochondrial disorders. It was first described in 1871 by the German ophthalmologist Theodor Leber and was subsequently named after him [4]. The disease is caused by mutations in mitochondrial DNA (mtDNA), leading to acute or subacute bilateral visual loss, usually in young adults, most frequently males. When mitochondria are affected due to complex I mutations, electron transport is impaired, and electron flow is reduced, resulting in increased levels of reactive oxygen species (ROS) and impaired ATP production [1,5]. Additionally, dysfunctional mitochondria are implicated in the triggering of apoptosis. The quality control of mitochondria requires a complex balance of mitobiogenesis and autophagy [6].

The symptoms arise due to selective degeneration and loss of retinal ganglion cells (RGCs). Studies show that the axons in the papillomacular bundle are the most susceptible to injury due to preferential involvement of smaller axons [7,8]. Subclinical ganglion cell dysfunction has been detected in asymptomatic carriers using pattern electroretinogram and structural changes on OCT [9]. Early symptoms include central or centrocecal scotoma, dyschromatopsia (colour desaturation), and defective pupillary light reflexes (in patients with asymmetric or monocular vision loss) [10]. During the acute phase, fundus examination may reveal vascular tortuosity and swelling (without leakage) of the retinal nerve fiber layer (RNFL) around the optic disc, optic disc hyperaemia, and peripapillary telangiectasias. Within weeks or months, the second eye is similarly affected, with an average interval of 6 weeks. A few months after onset, vision loss typically plateaus at or below 20/200 [11,12]. According to the latest research, LHON has four clinical stages: asymptomatic (mutation carriers), subacute (<6 months from onset), dynamic (6–12 months), and chronic (>12 months) [13].

The exact etiology and pathophysiology of the disease is still not fully understood. LHON mutations are passed on exclusively via the mother to her children, with no paternal contribution [14]. More than 95% of LHON cases are caused by three primary mutations: m.11778 G > A (ND4), m.3460 G > A (ND1), and m.14484 T > C (ND6), with m.11778 G > A being the most common [15]. These three variants affect genes that encode different mitochondrial subunits (m.3460 G > A: *MT-ND1*, m.11778 G > A: *MT-ND4*, and m.14484 T > C: *MT-ND6*) of complex I of the respiratory chain complex. However, not all individuals who harbor one or more of these primary variants develop a LHON phenotype [16]. The risk of visual loss is modulated by a combination of genetic and environmental factors, as well as anatomical, physiological, and hormonal influences. Only 50% of men and 10% of women who carry the LHON mtDNA mutation develop the optic neuropathy [17].

Pathogenic variants in the nuclear-encoded gene *DNAJC30* appear to interact with the mitochondrial ATP-synthase and have led to the stratification of LHON into maternal LHON (mtLHON) and autosomal recessive LHON (arLHON) [18]. A strong geographic accumulation of *DNAJC30* has been reported, with over 85% of the 29 families studied originating from Eastern Europe (Russia, Ukraine, Poland, and Romania) [19,20]. Stenton et al. reported that certain variants in the nuclear gene *DNAJC30* result in an autosomal recessive inherited form of LHON, with a notable geographic concentration of this gene in Eastern Europe [21]. Other recessive forms of LHON have recently been described, caused by mutations in the *MCAT*, *MECR*, and *NDUFS2* genes—variants of uncertain significance.

Current treatment options for LHON remain limited. In 2015, the European Medicines Agency approved Idebenone (Raxone^®^, Santhera Pharmaceuticals), Switzerland for the treatment of LHON in adolescents and adults, at a dosage of 900 mg/day divided into three doses. Oral idebenone is generally well tolerated, and reported side effects are rare [22]. Idebenone is a synthetic analogue of CoQ10 with antioxidant properties and the ability to penetrate mitochondrial membranes easily. It addresses impaired mitochondrial function in two ways: by bypassing the dysfunctional complex I of the mitochondrial respiratory chain, thereby restoring cellular ATP generation, and by acting as a potent antioxidant that reduces levels of ROS. Therefore, it may reactivate viable but inactive retinal ganglion cells (RGCs), promoting visual recovery in patients who have experienced vision loss [23]. Preclinical studies have confirmed a cell-line-specific increase in ATP production and reduced ROS levels in fibroblasts of LHON patients [24]. The efficacy of idebenone in LHON patients was first assessed in 2011, in the randomised, double-blinded, placebo-controlled RHODOS study conducted by Klopstock and co-authors. Based on their findings, idebenone appeared to prevent further visual loss in patients with discordant visual acuities, in contrast to the placebo group, whose visual acuities continued to deteriorate during the 24-week study period [25]. Since idebenone’s approval for commercial use, another multicentric study was initiated. This open-label, interventional Phase IV post-approval clinical trial was designed to assess the long-term efficacy and safety of idebenone in LHON patients [26]. The LEROS study confirmed the benefit of idebenone in LHON, including in the chronic phase (1–5 years since onset). Extending the treatment duration to 24 months maximized the rate of VA recovery. The authors recommend administering idebenone for more than 24 months, as vision may continue to deteriorate during the acute phase, while reaching the nadir, despite treatment initiation. However, in the subacute and dynamic phases, visual acuity can subsequently improve. Even if recent gene therapy research has showed promising results, further clinical trials are necessary to develop new treatments aimed at improving the quality of life of these patients [27].

The aim of this paper is to present our experience with the diagnosis and management of nine LHON patients harboring the three main mutation, as well as the *DNAJC30* mutation and a variant of uncertain significance (VUS). This is a prospective, observational, case-based study. We aimed to report our clinical outcomes and safety data, following long-term treatment with idebenone in clinical practice. The effect of idebenone treatment was analyzed based on the disease phase and the underlying genetic mutation.

## 2. Materials and Methods

We conducted a prospective, observational study that included 9 patients diagnosed with LHON between 2018 and 2024. Prior to 2017, no specific treatment was available in Romania for LHON patients. All patients were followed for a period of 18 months.

The inclusion criteria were the following: (1) bilateral acute or subacute, painless vision loss with central or paracentral scotomas; (2) swelling of RNFL or pallor of optic disc; and (3) presence of a confirmed LHON-associated DNA mutation—either mitochondrial or nuclear—verified by genetic testing. Exclusion criteria included the following: (1) high ametropia; (2) presence of glaucoma, ischemic optic neuropathy, or other optic nerve diseases; (3) macular degeneration or retinal vascular occlusion; and (4) severe systemic disease. All cases of partial optic atrophy of unknown etiology underwent comprehensive ophthalmic evaluation, followed by magnetic resonance imaging (MRI), Neuromyelitis Optica (NMO), and Myelin Oligodendrocyte Glycoprotein (MOG) level tests. After ruling out typical and atypical optic neuritis, as well as nutritional or toxic optic neuropathy, patients were tested for LHON mutations.

Patient gender, age, disease duration, and family history were recorded (Table 1).

All patients underwent a comprehensive ophthalmological examination, including bilateral best-corrected visual acuity (BCVA), visual field testing, intraocular pressure measurement, slit-lamp examination, fundus photography, and optical coherence tomography (OCT). This study followed the tenets of the Declaration of Helsinki, and all participants provided written informed consent.

All of our patients were eligible for idebenone treatment through the National Health Program for Rare Diseases, as they met the criteria for treatment initiation. In Romania, in order to receive treatment, patients must meet at least two criteria: one mandatory and one optional. The mandatory criterion is a positive genetic test confirming LHON. The optional criterion requires the presence of LHON-specific signs and symptoms that developed no more than five years prior to treatment initiation—such as decreased visual acuity below 1.0 logMAR within 12 months of clinical onset, dyschromatopsia, lack of response to corticosteroid therapy, or alterations in retinal ganglion cells (RGCs).

For the genetic testing, 5.0 mL of peripheral blood samples were collected in EDTA vacutainers. Polymerase chain reactions (PCR) were used to analyse the MT-ND1, MT-ND4, and MT-ND6 mitochondrial gene regions. The obtained sequences were then compared with reference sequences. Four patients carried the 11778 mutation, one carried the 14484 mutation, two carried the 3460 mutation, one carried the nuclear *DNA JC30* mutation, and one carried a variant of uncertain significance MT ATP6. Three eyes were analysed in the acute phase (less than 6 months after symptoms onset), one in the dynamic phase (6 to 12 months after symptom onset), and five in the chronic phase (more than 12 months after symptom onset). The time of symptom onset was assessed by the patient’s history.

The BCVA was generally assessed using Early Treatment Diabetic Retinopathy Study (ETDRS) charts, with values expressed in logarithm of the minimum angle of resolution (logMAR), or converted from Snellen notation to logMAR for analysis.

Tomographic imaging of both eyes was performed using high-definition spectral-domain OCT (REVO FC, OPTOPOL). Each subject had both eyes scanned using two standard acquisition protocols: macular cube 512 × 128 and optic disc cube 200 × 200. The quality of the obtained images was assessed by evaluation of the signal strength. For the RNFL analysis, the scanned optic disc cube 200 × 200 was used. Subsequently, a recognition algorithm detected the inner and outer borders of the RNFL. We noted RNFL thickness at specific locations around the optic nerve: temporal, superior, nasal, and inferior. For the ganglion cell layer (GCL) analysis, the macular cube 512 × 128 centered in the fovea was used. A different recognition algorithm was applied to detect the outer border of the RNFL and the inner plexiform layer. The average RNFL and GCL thickness values, measured in micrometers (μm), were used for statistical analysis. Normal RNFL thickness was defined as 103 +/− 10.90 µm, and normal GCL thickness was defined as between 68 and 74 µm.

Patients were treated with oral idebenone at a dose of 900 mg/day, divided into 3 doses. In Romania, idebenone administration cannot exceed 36 months, and if no improvement is observed, treatment should be discontinued after 24 months.

We evaluated treatment efficacy using the criteria of clinically relevant recovery (CRR) and clinically relevant stabilization (CRS). CRR was defined as improvement of at least 10 ETDRS letters (2 lines on ETDRS chart or 0.2 logMAR units) for on chart patients, with visual acuity 1.0 logMAR or less, or an improvement from off-chart (>1.69 logMAR) to on-chart by at least 5 ETDRS letters. CRS was defined in patients with visual acuity less than 1.0 logMAR at baseline, who maintained the same visual acuity at the last visit. Clinically relevant benefit (CRB) was defined as achieving either CRR and/or CRS.

## 3. Results

Eighteen eyes from nine patients were investigated and visual acuity (VA), pRNFL, and GCL were assessed at baseline. After treatment with idebenone, VA was assessed at 3, 6, 9, 12, and 18 months, and pRNFL and GCL were assessed at 6, 12, and 18 months. The mean age of the patients was 29.67 ± 8.53 years, and the mean age at disease onset was 29.67 ± 8.79 years.

Of the 18 eyes, 6 were in the acute phase, 2 in the dynamic phase, and 10 in the chronic phase; 8 eyes had the 11778 G > A mutation, 4 eyes had the 3460 G > A mutation, 2 eyes had the 14484 T > C mutation, 2 eyes had the *DNA JC 30* mutation, and 2 eyes had the *MT ATP 6* mutation.

**Figure 1:**
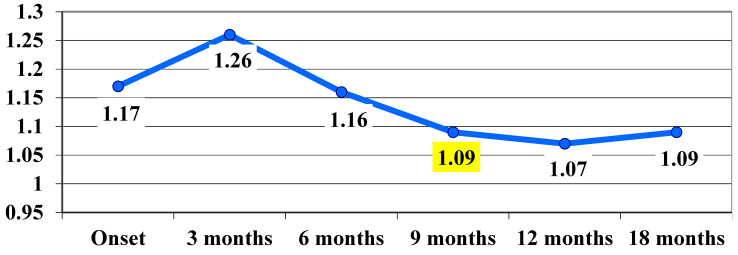
Visual acuity, VA, evolution in logMAR during follow-up (average values). The highlighted values are statistically significant.

The t Student calculation for highlighting the significance of differences between the average values in VA at the transition from one stage of the assessment to another showed that, between the 6-month and 9-month assessments, the difference was significant, with the average value decreasing significantly from 1.16 logMAR ± 0.59 at 6 months to 1.09 logMAR ± 0.56 at 9 months, with t(17) = 2.2 and *p* = 0.04 (Table 2, Figure 1).

It can be said that, following the treatment administered, at 9 months there was a significant improvement in VA, which remained at the same level at 12 and 18 months.

**Figure 2:**
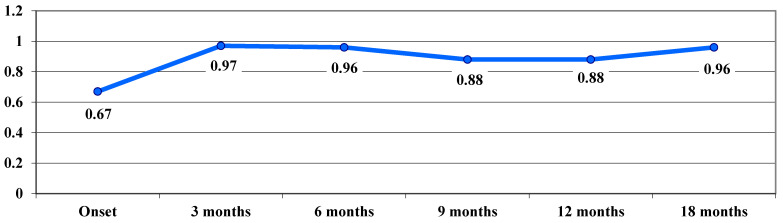
Evolution of visual acuity, VA, in logMAR in the acute phase.

Regarding the evolution of VA in the acute phase, a deterioration of VA was observed from 0.67 logMAR at baseline to 0.97 logMAR at 3 months, with a slight improvement in VA observed at 9 months—0.88 logMAR (Figure 2).

**Figure 3:**
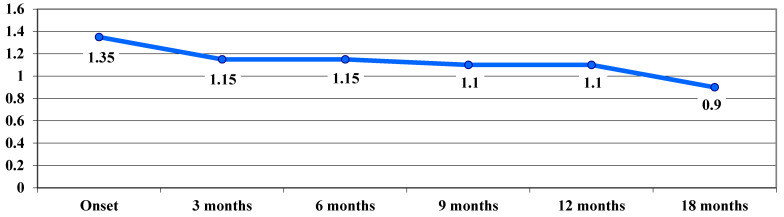
Evolution of visual acuity, VA, in logMAR in the dynamic phase.

VA in the dynamic phase showed a trend of continuous improvement during follow-up, from a VA average value of 1.35 logMAR at baseline to 0.9 logMAR after 18 months, with no significant differences between stages (Figure 3).

**Figure 4:**
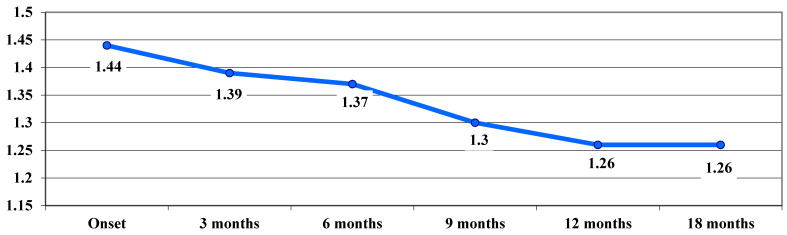
Evolution of visual acuity, VA, in logMAR in the chronic phase.

In the chronic phase, following treatment, an improvement in VA was observed, with average VA values showing a downward trend from one stage of the examination to the next, from 1.44 logMAR at baseline to 1.26 logMAR at 12 and 18 months, with no significant differences between stages (Figure 4).

**Figure 5:**
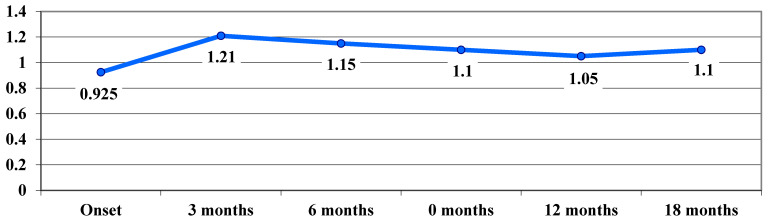
Evolution of visual acuity, VA (average values), in logMAR in cases with the 11778 G > A mutation.

Sub-analyses by mutation showed a consistent treatment benefit over time in eyes with the m.11778 G > A mutation. In the acute phase, we noticed a worsening of the VA, from 0.9 logMAR at baseline to 1.21 logMAR at 3 months, while reaching the nadir. At 12 and 18 months, treated eyes improved from 1.21 logMAR at 3 months to 1.11 logMAR. (Figure 5).

**Figure 6:**
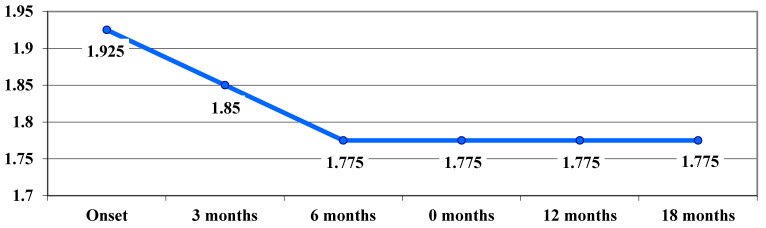
Evolution of visual acuity, VA (average values), in logMAR in cases with the 3460 G > A mutation.

The benefit of treatment was also observed in patients with the 3460 G > A mutation in the chronic phase. Average VA values showed a downward trend (not statistically significant) up to 6 months (from 1.925 logMAR at baseline to 1.85 logMAR at 3 months and 1.775 logMAR at 6 months), after which they remained constant up to 18 months (Figure 6).

In cases with the 14484 T > C mutation, the average VA values tended to decrease, from 1.6 logMAR at baseline to 1.5 logMAR at 3 and 6 months and then to 1.3 logMAR at 9 months, remaining constant until 18 months, with a beneficial effect of treatment also observed here. In cases with the *DNAJC30* mutation, a significant improvement in VA was observed, with a downward trend in average VA values from 0.6 logMAR at baseline and 3 months to 0.15 logMAR at 6 months, and then to 0.05 logMAR at 9 months, remaining constant until 18 months. In cases with the MT ATP 6 mutation, the VA average value increased insignificantly from 0.45 logMAR at baseline to 0.55 logMAR at 3 months, after which it remained constant until 18 months at an average value of 0.5 logMAR (Table 3).

**Figure 7:**
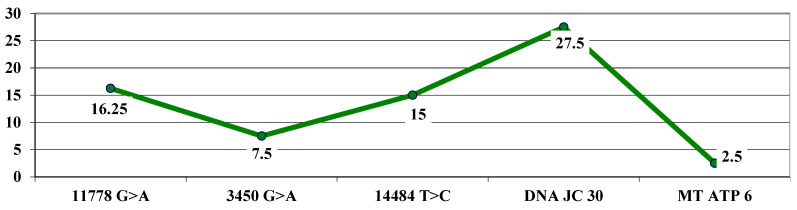
Highest letter gain (average values) based on mutations.

The highest average letter gain was observed in association with the *DNAJC30* mutation (27.5 letters), followed by the association with the 11778 G > A mutation (16.25 letters), 14484 T > C (15 letters), 3460 G > A (7.5 letters), and, the lowest gain, at the MT ATP 6 mutation (2.5 letters) (Figure 7).

The *t*-test calculation (independent samples) showed a significantly higher average maximum letter gain for the 11778 G > A mutation (16.25 letters) compared to 7.5 for the 3460 G > A mutation, with t = 2.10 and *p* = 0.03.

Next, we will classify the patients in on-chart and off-chart, according to VA. We defined on-chart patients the patients with a VA between 0 and 1 logMAR and off-chart patients the patients with a VA between 1 and 2 logMAR.

At the beginning of the study, there were 11 off-chart cases (61.11%); after 3 months, the frequency decreased to 10 cases (a decrease of 5.55%), which remained stable at 6 and 9 months, and then decreased to 8 cases in month 12 (a further decrease of 11.11%) and remained stable in month 18 (Table 4).

The frequency of on-chart cases increased from 38.89% at baseline to 55.56% in month 12 (an increase of 16.67%) and remained at the same level through month 18.

**Figure 8:**
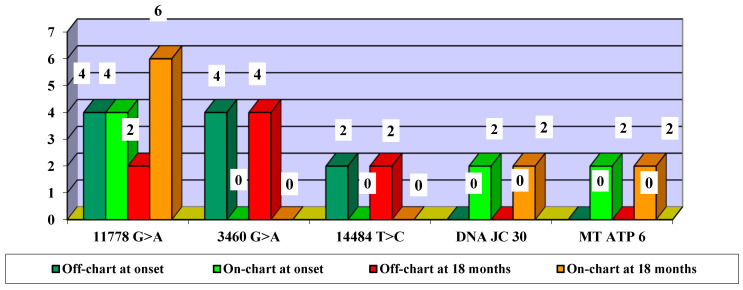
Evolution of visual acuity VA off-chart vs. on-chart cases, according to mutations—number of eyes.

In terms of mutation evolution, the largest increase in the frequency of on-chart cases from baseline to month 18 occurred in association with the 11778 G > A mutation (an increase from two cases at baseline to six cases at month 18). The largest decrease in off-chart cases occurred in cases associated with the 3460 G > A mutation (from 4 cases at baseline to 0 cases at month 18), followed by the association with the 14484 T > C mutation (from 2 cases at baseline to 0 cases at month 18) (Figure 8).

Using OCT, we evaluated pRNFL and GCL. Regarding pRNFL, Student t calculation showed that, between the initial pRNFL assessment and the 6-month assessment, the difference was significant, with the average value decreasing significantly from 100.83 µ ± 30.2 at baseline to 96.7 µ ± 24.8 at 6 months, with t(17) = 2.8 and *p* = 0.013. A significant decrease in pRNFL was found at 6 and 12 months, which continued at 18 months. Compared to the average value at 12 months, the average GCL decreased significantly at 18 months. Between the 12-month and 18-month assessments, the difference was significant: from 60.56 µ ± 3.43, the average value decreased significantly to 59.56 µ ± 3.31 at 18 months, with t(17) = 4.1 and *p* < 0.001.

We then evaluated the effectiveness of treatment with idebenone. We classified patients into CRB (clinically relevant benefit) and CRW (clinically relevant worsening). CRB included patients who showed CRR (clinically relevant recovery) and/or CRS (clinically relevant stabilization). CRR was defined as improvement of at least 10 ETDRS letters (2 lines on ETDRS chart or 0.2 logMAR units) for on chart patients, with visual acuity 1.0 logMAR or less or improvement from off-chart (>1.69 logMAR) to on-chart by at least 5 ETDRS letters. CRS was defined in patients with visual acuity less than 1.0 logMAR at baseline, who maintained the same visual acuity at the last visit.

**Figure 9:**
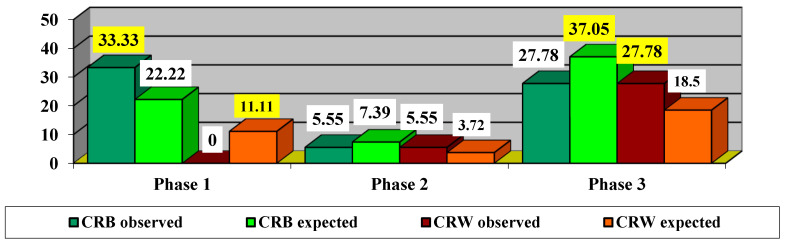
Impact of treatment depending on phase. CRB—clinically relevant benefit =clinically relevant recovery (CRR) or/and clinically relevant stabilization (CRS). CRW—clinically relevant worsening. The values highlighted in yellow represent statistically significant values.

After treatment with idebenone, 12 eyes (66.67%) were classified as CRB and 6 eyes (33.33%) as CRW (Figure 9). The values highlighted in yellow represent statistically significant values.

Since the χ^2^ value with Yates correction = 4.5 is significant at *p* = 0.034, it proves that there are significant differences in the impact of treatment depending on the phase. Thus, it was found that, in the acute phase, there were more CRB cases observed (33.33% compared to 22.22% expected) and fewer CRW cases observed (0% compared to 11.11% expected). In the dynamic phase, there were slightly fewer CRB cases observed (5.55% compared to 7.39% expected) and slightly more CRW cases observed (5.55% compared to 3.72% expected). In the chronic phase, fewer CRB cases were observed (27.78% compared to 37.05% expected), and more CRW cases were observed (27.78% compared to 18.5% expected).

We continued to evaluate the efficacy of idebenone treatment according to mutation (Table 5).

Since the χ^2^ value with Yates correction = 11.8125 is significant at *p* = 0.0006, we can consider that there are significant differences in the impact of treatment depending on mutations. Thus, it can be observed that in mutation 11778 G > A there were more CRB cases observed (38.89% compared to 29.61% expected) and fewer CRW cases observed (5.55% compared to 14.83% expected). In mutation 3460 G > A, fewer CRB cases were observed (0% compared to 14.83% expected), and more CRW cases were observed (22.22% compared to 7.39% expected). In the 14484 T > C mutation, there were slightly fewer CRB cases observed (5.55% compared to 7.39% expected) and slightly more CRW cases observed (5.55% compared to 3.72% expected). In the *DNAJC30* mutation, there were more CRB cases observed (11.11% compared to 7.39% expected) and fewer CRW cases observed (0% compared to 3.72% expected). In the MT ATP 6 mutation, there were more CRB cases observed (11.11% compared to 7.39% expected) and fewer CRW cases observed (0% compared to 3.72% expected).

**Figure 10:**
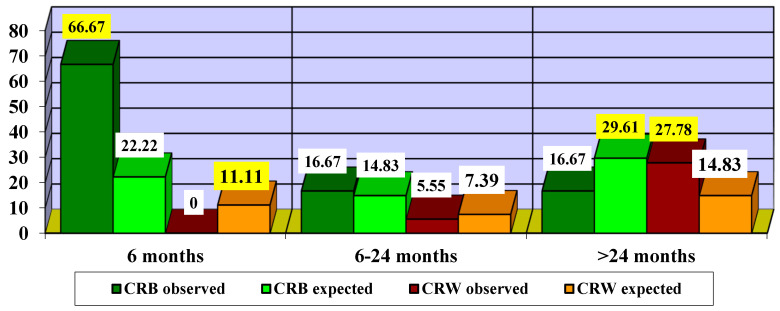
Impact of treatment according to time to treatment initiation. CRB—clinically relevant benefit; CRW—clinically relevant worsening. The highlighted values are statistically significant.

Next, we attempted to find out the optimal time to start treatment (Figure 10).

Since the χ^2^ value = 6.1875 is significant at *p* = 0.0129, we can consider that there are significant differences in the impact of treatment according to the different times of treatment initiation. Thus, it can be observed that initiation of treatment in the first 6 months led to more CRB cases (66.67% compared to 22.22% expected) and fewer CRW cases (0% compared to 11.11% expected). Initiating treatment with idebenone between 6 and 24 months led to slightly more CRB cases (16.67% compared to 14.83% expected) and slightly fewer CRW cases (5.55% compared to 7.39% expected). In patients who started treatment after 24 months, fewer cases of CRB (16.67% compared to 29.61% expected) and more cases of CRW (27.78% compared to 14.83% expected) were observed.

We also noted the adverse effect of idebenone in our patients. The majority of treatment-emergent adverse events were mild, including headache in three out of nine patients, diarrhea in two patients, and nausea in one patient.

## 4. Discussion

In our prospective study, we investigated the long-term visual outcomes in LHON patients who received idebenone treatment for a period of 18 months. Idebenone is the first approved treatment for LHON; however, its benefit compared to the natural history of the disease remains debated. Many studies suggested that idebenone is an effective and safe treatment for these patients [25,26,28]. Idebenone reduces autophagy and apoptosis in LHON fibroblasts and normalises mtROS production and membrane potential. The reduction in ROS may promote remyelination of surviving denuded axons, potentially restoring the viability of RGCs [29,30]. However, spontaneous recovery has occasionally been reported in LHON patients [31]. Although visual function improvement typically occurs within the first months after reaching the nadir, it is less likely to occur years later. Therefore, we cannot exclude the possibility that visual improvement in some patients was attributable to the natural course of the disease. In our study, the frequency of on-chart cases increased from 38.89% at baseline to 55.56% at 12 months.

Regarding the natural history of visual outcomes, Sadun et al. (2004) studied a Brazilian cohort of 20 LHON subjects, with a mean age of onset 26.1 ± 10 years. None of the patients reported visual recovery, and central vision function was severely impaired in all eyes, with a mean final VA of 2.04 logMAR [32].

In our study, following treatment administration, a significant improvement in VA was observed at 9 months, which remained stable at 12 and 18 months. In the acute phase, VA deteriorated from 0.67 logMAR at onset to 0.97 logMAR at 3 months, followed by a slight improvement to 0.88 logMAR at 9 months. In the dynamic phase, VA showed a continuous improvement trend during the follow-up period, from a mean visual acuity of 1.35 logMAR at onset, to 0.9 logMAR at 18 months. In the chronic phase, following the treatment administered, VA improved, with average values decreasing progressively from one stage to another after examination, from 1.44 logMAR at onset to 1.26 logMAR at 12 and 18 months.

In 2014, Romero et al. described a cohort of 21 Chilean LHON patients harboring the mt.11778A, with age at onset ranging from 3 to 53 years. The mean final VA was 1.8 logMAR [33].

In 2019, Silva et al. studied a subgroup of 61 untreated subjects with m.11778G.A mutation, including children as young as 6 years. The results showed a CRR in 15% of patients from baseline and 28% of subjects from nadir [34]. In 2020, Moon et al. reported a cohort of 40 LHON patients, 16 of whom were treated with idebenone. Visual recovery was noted in 23.2% of eyes in patients with the m.11778G.A mutation [35].

In our study, we also observed a consistent treatment benefit over time in eyes harbouring the m.11778 G > A mutation. In the acute phase, visual acuity (VA) worsened from baseline to 3 months, while reaching the nadir. However, by 12 and 18 months, treated eyes had an improvement in VA. As some authors reported spontaneous recovery in some natural history patients, we should take in consideration that the visual recovery in some of our patients cannot be entirely attributed to idebenone treatment alone [33,34,35].

Although the most powerful predictor of visual outcome remains the mtDNA genotype—with the m.11778G.A mutation associated with the worst prognosis—young age at onset is correlated with a better chance of visual recovery. In our study, the 17-year-old patient demonstrated the most favorable prognosis. Newman et al. reported that, among adult patients with m.11778G.A mutation, recovery of vision from nadir occurs in fewer than 20% of patients, and final visual acuities better than 20/200 are rare [36].

Our findings also suggest that the impact of treatment varies significantly depending on the disease phase. Thus, in the acute phase, more cases of clinically relevant benefit (CRB) were observed than expected (33.33% versus 22.22% expected) and fewer CRW cases observed (0% versus 11.11% expected). In the chronic phase, fewer CRB cases were observed (27.78% versus 37.05% expected) while CRW cases were more frequent (27.78% versus 18.5% expected).

Our results are consistent with those of previous studies. RHODOS is the first ever completed, randomized, placebo-controlled trial in LHON, including 85 LHON patients across three centers (Munich, Newcastle, and Montreal) [25]. Although this study did not meet the primary endpoint, all pre-specified secondary visual acuity endpoints, subgroup, and responder analyses indicated a beneficial effect of idebenone. Based on their findings, idebenone appeared to prevent further visual loss in patients with discordant visual acuities, in contrast to the placebo group, whose visual acuities continued to deteriorate over the 24-week study period. Notably, one in three patients in the idebenone group experienced a CRR, and 28% of patients who were “off-chart” at baseline achieved a CRR in the idebenone-treated group.

We can consider that there are significant differences in the impact of the treatment depending on the mutations. Thus, it can be seen that, for the 11778 G > A mutation, more CRB cases were observed (38.89% compared to 29.61% expected) and fewer CRW cases observed (5.55% compared to 14.83% expected). For the 3460 G > A mutation, fewer CRB cases were observed (0% compared to 14.83% expected) and more CRW cases observed (22.22% compared to 7.39% expected). For the 14484 T > C mutation, slightly fewer CRB cases were observed (5.55% compared to 7.39% expected), and slightly more CRW cases were observed (5.55% compared to 3.72% expected). In the *DNAJC30* mutation, more CRB cases were observed (11.11% vs. 7.39% expected), and fewer CRW cases were observed (0% vs. 3.72% expected). Our results are similar to those of other studies.

The LEROS Study—an open-label, phase IV post-approval clinical trial to assess the efficacy and safety of idebenone in LHON patients—is a controlled, multicentric study that included adult patients from 29 centers in 10 countries over a period of 5 years. Patients received idebenone at a dose of 900 mg/day. The results were compared with a retrospective cohort of untreated patients [37]. Although not statistically significant, CRR rates in the dynamic stage indicated a positive treatment effect at 12 months (33.1% treated vs. 18.1% untreated) and 24 months (47.9% vs. 33.3%). The frequency of CRR from baseline at 12 months was significantly higher in treated chronic eyes versus matched NH eyes (50.3% vs. 38.6%). This was maintained at 24 months (49.1% vs. 37.6%). The treatment effect varied depending on disease phase and the causative mtDNA mutation, with a consistent benefit observed in patients with the most common m.11778 G > A mutation across all phases, and in patients with the m.14484 T > C mutation in the chronic phase.

In our study, we observed a higher average maximum letter gain in patients having significantly higher levels at the mutation 11778 G > A (16.25 letters) compared to 7.5 at the mutation 3460 G > A. The benefit of the treatment was also observed in patients with the 3460 G > A mutation in the chronic phase. The average values of VA in logMAR showed a tendency to decrease up to 6 months, after which it remained constant until 18 months. In cases with the mutation 14484 T > C, average VA values also decreased at 6 months and 9 months and remained constant through 18 months, indicating a beneficial treatment effect. Further study of idebenone use in patients carrying the m.3460 G > A mutation is needed to clarify treatment benefits. In cases with the *DNAJC30* mutation, a significant improvement in VA was observed. This is consistent with other studies that have shown a better prognosis in arLHON patients [38,39].

Another study, PAROS, was a phase IV post-authorization study with the primary objective of further evaluating the long-term safety profile of idebenone in the treatment of LHON patients in routine clinical care [40]. A total of 58.0% of patients experienced 382 treatment-emergent adverse events, and 22.3% of patients experienced 82 adverse drug reactions. The majority of treatment emergent adverse events were considered mild to moderate in intensity—headache, diarrhea, nasopharyngitis, nausea, cough, and abdominal pain. Related-treatment, emergent adverse events occurred more frequently in idebenone-naïve patients from baseline to the last visit, with the highest incidence in the first year after baseline and a stable rate from the third year onward.

In our study, the majority of treatment-emergent adverse events were also mild, including headache in three out of nine patients, diarrhea in two patients, and nausea in one patient.

The introduction of OCT has enhanced our understanding of the structural changes in the macula and optic nerve that occur in LHON patients. A recent study suggests that RNFL thickness at a given moment can help determine the stage of LHON [41]. Balduci et al. observed that, in the acute phase, OCT shows thinning of the peripapillary RNFL in the inferior and temporal quadrants, as well as thinning of the nasal macular GC-IPL, reflecting early involvement of the papillomacular bundle. Therefore, early ganglion cell loss may partially account for the absence of a significant association between visual acuity and GC-IPL thickness, as most patients already exhibit substantial ganglion cell loss in these regions before initiating idebenone treatment [42].

Regarding OCT measurement, our study highlighted a significant difference in pRNFL thickness between the initial evaluation and the 6-month follow-up, with the mean value decreasing from 100.83 µm ± 30.2 at baseline to 96.7 µm ± 24.8 at 6 months. A significant decrease in pRNFL was also observed at 6 and 12 months, which continued at 18 months. This is consistent with the findings of Miao et al., who reported that in the chronic stage, pRNFL thickness was significantly reduced compared to controls, suggesting severe damage to the function and structure of RGCs [43]. On the other hand, Wang et al. found that, in LHON patients with disease onset less than 3 months, average pRNFL thickness did not change significantly [44]. A study of seven LHON patients with recent onset found that RNFL thinning did not correlate with final visual acuities [45]. In our study, the average GCL thickness significantly decreased at 12 and 18 months compared to baseline. Between the 12-month evaluation and the 18-month evaluation, the difference was significant: from 60.56 µ ± 3.43, the average value significantly decreased to 59.56 µ ± 3.31 at 18 months.

This finding aligns with previous studies showing that, in addition to pRNFL thinning, the macular RGC layer is also significantly reduced in affected individuals [46,47]. Barboni et al. reported that RNFL thickening initially appears in the temporal and inferior quadrants. The late involvement of both superior and nasal quadrants suggests dynamic progression during the acute stage [48]. Therefore, GC-IPL thickness may serve as a more reliable marker than pRNFL thickness. Early detection of GC thinning alongside RNFL swelling on optical coherence tomography (OCT) could facilitate prompt diagnosis and timely intervention [49,50].

## 5. Conclusions

Studying rare diseases presents significant challenges. Clinical trials are usually difficult to conduct due to the low prevalence of these diseases in the general population.

Our paper demonstrates the benefit of idebenone treatment in improving visual acuity in patients with Leber hereditary optic neuropathy. In our study, treatment response was observed even in cases of severe visual impairment; however, early initiation of therapy was associated with a higher likelihood of response. The small sample size is a limitation, but it reflects the rarity of the disease. Identifying effective therapies for such patients remains crucial. A treatment duration of at least 18 months is needed to maximize the probability of clinically relevant benefit.

We highlighted the importance of long-term treatment, emphasizing that extended administration is key to achieving favorable outcomes. The safety results that we observed were consistent with the known safety profile of idebenone.

Several important questions remain and warrant further clinical investigation: the optimal duration of treatment, the maximum therapeutic window from symptom onset, the identification of biomarkers predictive of treatment response, and the potential role of idebenone prophylaxis in asymptomatic mutation carriers.

LHON is a complex condition that demands ongoing research to develop effective therapies aimed at minimizing significant vision loss.

## Figures and Tables

**Table 1:** Patients’ demographic data.

Patient	Gender	Age at Onset	Duration Between Symptom Occurrence and Diagnosis	Mutation	Family History
1	M	22	6 months	11778 G > A	Brother positive, but asymptomatic
2	M	30	24 months	11778 G > A	Cousin lost vision, not diagnosed
3	M	27	6 months	11778 G > A	No positive family history
4	M	35	60 months	3460 G > A	Brother positive, symptomatic
5	M	37	60 months	3460 G > A	Brother positive, symptomatic
6	M	32	60 months	14484 T > C	No positive family history
7	M	20	12 months	11778 G > A	Brother positive, symptomatic
8	M	17	6 months	*DNAJC30*	Brother positive, but asymptomatic
9	M	22	48 months	*MT ATP6*	No positive family history

**Table 2:** Average visual acuity, VA, values in logMAR during follow-up. The highlighted values are statistically significant.

Indicators	Onset	At 3 Months	At 6 Months	At 9 Months	At 12 Months	At 18 Months
**Average visual acuity, VA, in logMAR**	1.17 logMAR	1.26 logMAR	**1.16** logMAR	***1.09↓*** logMAR	1.07 logMAR	1.09 logMAR
**Standard deviation**	0.63	0.53	0.59	0.56	0.55	0.57
**t(17)**		1.2	2	2.2	0.7	0.5
***p***		0.25	0.06	**0.04**	0.47	0.65

**Table 3:** Visual acuity, VA, values in logMAR in cases with 14484 T > C, *DNAJC30* and MT ATP 6 mutations. For each mutation, the first column shows the values of VA for each eye and the second column, the average values, at the onset and follow-up visits.

Determination	14484 T > C Mutation		*DNAJC30* Mutation		MT ATP 6 Mutation	
	Values logMAR	Average logMAR	Values logMAR	Average logMAR	Values logMAR	Average logMAR
Onset	1.7; 1.5	1.6	1; 0.2	0.6	0.5; 0.4	0.45
At 3 months	1.7; 1.3	1.5	1; 0.2	0.6	0.7; 0.4	0.55
At 6 months	1.7; 1.3	1.5	0.3; 0	0.15	0.6; 0.4	0.5
At 9 months	1.3; 1.3	1.3	0.1; 0	0.05	0.6; 0.4	0.5
At 12 months	1.3; 1.3	1.3	0.1; 0	0.05	0.6; 0.4	0.5
At 18 months	1.3; 1.3	1.3	0.1; 0	0.05	0.6; 0.4	0.5
Maximum letter gain	15; 15	15	45; 10	27.5	5; 0	2.5

**Table 4:** Frequency of off-chart vs. on-chart cases at onset and during treatment.

VA	Onset		At 3 Months		At 6 Months		At 9 Months		At 12 Months		At 18 Months	
	N	%	N	%	N	%	N	%	n	%	n	%
**Off-chart**	11	61.11	10	55.56	10	55.56	10	55.56	8	44.44	8	44.44
**On-chart**	7	38.89	8	44.44	8	44.44	8	44.44	10	55.56	10	55.56

**Table 5:** Frequency of cases after treatment impact according to mutations. CRB—clinically relevant benefit = clinically relevant recovery (CRR) or/and clinically relevant stabilization (CRS). CRW—clinically relevant worsening.

Mutations	CRB Cases				CRW Cases			
	Observed		Expected		Observed		Expected	
	N	%	N	%	N	%	N	%
11778 G > A	7	38.89	5.33	29.61	1	5.55	2.67	14.83
3460 G > A	0	0	2.67	14.83	4	22.22	1.33	7.39
14484 T > C	1	5.55	1.33	7.39	1	5.55	0.67	3.72
DNA JC 30	2	11.11	1.33	7.39	0	0	0.67	3.72
MT ATP 6	2	11.11	1.33	7.39	0	0	0.67	3.72

## Data Availability

Data analyzed in our article is contained within this published paper.

## Abbreviations

The following abbreviations are used in this manuscript:

LHON	Leber’s hereditary optic neuropathy
DOA	dominant optic atrophy
VA	visual acuity
mtDNA	mitochondrial DNA
ROS	reactive oxygen species
OCT	optical coherence tomography
RNFL	retinal nerve fiber layer
arLHON	autosomal recessive LHON
mtLHON	maternal LHON
VUS	variant of uncertain significance
RGC, GCL	retinal ganglion cells layer
CRB	clinically relevant benefit
CRR	clinically relevant recovery
CRS	clinically relevant stabilisation
CRW	clinically relevant worsening
